# Physiological, cytological, and reproductive hormone characterization of estrus in Pasundan heifers synchronized with double injection of prostaglandin F2α

**DOI:** 10.14202/vetworld.2025.1357-1364

**Published:** 2025-05-31

**Authors:** Rini Widyastuti, Nena Hilmia, Diky Ramdani, Rahmat Hidayat, Iman Hernaman, Andre Rivanda Daud, Ken Ratu Ghazirah Alhuur, Matni Syifa Bayani, Rangga Setiawan, Sigit Prastowo, Santoso Santoso, Vidi Wulandari

**Affiliations:** 1 Department of Animal Production, Faculty of Animal Husbandry, Universitas Padjajaran, Sumedang, West Java, 45363, Indonesia; 2 Department of Animal Nutrition and Feed Technology, Faculty of Animal Husbandry, Universitas Padjadjaran, Sumedang, West Java, 45363, Indonesia; 3 Department of Socio-economics of Livestock Development, Faculty of Animal Husbandry, Universitas Padjajaran, Sumedang, West Java, 45363, Indonesia; 4 Department of Animal Science Faculty of Animal Science, Universitas Sebelas Maret, Surakarta, Indonesia; 5 Research Center for Animal Husbandry, National Research and Innovation Agency, Cibinong Science Center, Jl. Raya Jakarta, Bogor, West Java, 16915, Indonesia; 6 UPTD Center for Artificial Insemination of Beef Cattle Breeding and Development Center, Ciamis, West Java, Indonesia

**Keywords:** estrus synchronization, fixed-time artificial insemination, Pasundan cattle, prostaglandin F2α, reproductive hormones, vaginal cytology

## Abstract

**Background and Aim:**

Pasundan cattle, a native Indonesian breed with valuable reproductive traits, face population decline due to limited conservation efforts. Estrus synchronization using prostaglandin F2α (PGF2α) is a viable strategy to support genetic improvement and sustainable breeding. This study aimed to evaluate the physiological, cytological, and hormonal responses associated with estrus synchronization in Pasundan heifers following a double-injection protocol of PGF2α.

**Materials and Methods:**

Eighteen healthy Pasundan heifers (2.0–2.5 years old; body condition score 3) received two intramuscular PGF2α injections 11 days apart. Estrus signs were assessed through vulva morphology, cervical mucus viscosity, and vaginal electrical resistance (VER) on days 0, 4, 5, 11–15. Vaginal cytology was conducted to classify epithelial cells, and blood samples were analyzed for follicle-stimulating hormone (FSH), progesterone, and estradiol through enzyme-linked immunosorbent assay. Data were analyzed using one-way analysis of variance with Tukey’s *post hoc* test (p < 0.05).

**Results:**

The peak estrus response occurred on day 14 post-initial injection, marked by maximal vulvar swelling (7.27 ± 1.15 cm), highest mucus viscosity (14.9 ± 3.00 mm), and lowest VER (198.67 ± 29.61 Ohms). Cytologically, superficial and keratinized epithelial cells dominated (64.22%), indicating estrus. Hormonal assays revealed elevated FSH (5.08 mIU/mL) and estradiol (0.214 pg/mL), alongside a nadir in progesterone (0.162 ng/mL). Estrus was observed in 88.89% of heifers on day 14.

**Conclusion:**

Double-injection PGF2α effectively synchronized estrus in Pasundan heifers, with day 14 being optimal for fixed-time artificial insemination (FTAI). The synchronization protocol demonstrated clear correlations between physical, cytological, and hormonal parameters. This protocol provides a reliable basis for reproductive management in Pasundan cattle, facilitating conservation and productivity. Future studies should assess conception outcomes post-FTAI to validate long-term reproductive efficiency.

## INTRODUCTION

Pasundan cattle, an indigenous breed originating from the southern coastal region of West Java and the northern Priangan area of Indonesia, possess notable genetic potential owing to their superior reproductive performance, robust resistance to parasitic infections, and adaptability to challenging environmental cond-itions [1]. Despite these advantages, the population of Pasundan cattle has been declining steadily each year, raising concerns about the breed’s long-term viabi-lity [2]. Consequently, strategic conservation efforts are urgently required to safeguard and propagate these valuable genetic traits. Assisted reproductive techn-ologies (ARTs), encompassing sperm cryopreservation, sex-sorting, artificial insemination (AI), *in vitro* embryo production, and estrus synchronization, represent the most effective tools to support such conservation and genetic improvement programs [3].

Among the various ART approaches, estrus synchronization has emerged as a widely adopted and practical strategy to enhance reproductive efficiency, particularly under semi-intensive management systems where estrus detection poses a significant challenge. A key hormonal agent used in synchronization protocols is prostaglandin F2α (PGF2α), which facilitates luteolysis and regression of the corpus luteum, thereby initi-ating estrus [4–8]. PGF2α can be administered through either single or double injections, with the latter method demonstrating superior outcomes in terms of synchronization success. Comparative studies have shown that the double-injection protocol leads to a more pronounced reduction in circulating progesterone levels and more efficient luteolysis, thereby improving the rate and uniformity of estrus expression [9–12]. These findings have been corroborated in both Bos indicus breeds, such as Boran cattle [13], and in dairy cows [14], where the double-injection protocol consistently yielded higher estrus response rates than the single-injection approach.

Although estrus synchronization using PGF2α has been extensively studied in various cattle breeds, including Bos indicus and dairy cattle, limited information exists on its physiological, cytological, and hormonal impacts in indigenous Indonesian breeds such as Pasundan cattle. Most available studies have focused predominantly on estrus response rates or conception outcomes, without providing a comprehensive analysis of the underlying estrus indicators that could optimize fixed-time AI (FTAI). Given the genetic uniqueness and reproductive potential of Pasundan cattle, the lack of data on breed-specific estrus characteristics – such as changes in vulvar morphology, vaginal cytology, and hormonal dynamics – represents a critical gap. Mor-eover, no previous studies have clearly defined the optimal timing for FTAI in Pasundan cattle follo-wing a double injection PGF2α-based synchronization protocol.

This study aimed to characterize the physiological, cytological, and reproductive hormonal responses associated with estrus in Pasundan heifers following a double-injection protocol of PGF2α. By evaluating vulvar swelling; vaginal mucus viscosity; vaginal electrical resistance (VER); epithelial cell composition; and serum levels of FSH, estradiol, and progesterone across multiple time points, the study sought to determine the peak estrus period. The ultimate objective was to identify the optimal time for FTAI in Pasundan cattle, thereby contributing to more effective and targeted reproductive management strategies for this declining indigenous breed.

## MATERIALS AND METHODS

### Ethical approval

All animal procedures in this study were cond-ucted in accordance with ethical standards and were approved by the Universitas Padjadjaran Health Research Ethics Committee (Approval No. 1398/UN6.KEP/EC/2023).

### Study period and location

The study was conducted during June and July 2024 at the Beef Cattle Teaching Farm, Faculty of Animal Husbandry, Universitas Padjadjaran, located in Sumedang Regency, West Java, Indonesia

### Experimental animals

The study involved 18 (n = 18) Pasundan heifers, aged between 2.0 and 2.5 years, with a body condition score (BCS) of 3 on a 1–5 scale [15]. Before inclusion in the study, all animals underwent rectal palpation to assess reproductive status and to confirm the absence of pregnancy.

### Estrus synchronization protocol

Estrus synchronization was performed using double injection of PGF2α intramuscularly (Synchrom-ate, Bremer Pharma GmbH, Warburg, Ger-many; 0.25 mg/mL Cloprostenol) at a dosage of 1 mL per animal. The injections were administered 11 days apart, with the first designated as day 0 (D0) and the second as day 11 (D11). Estrus parameters – including vulvar dimensions, cervical mucus viscosity, VER, vaginal cytology, and reproductive hormone levels – were monitored on D0, D4, D5, D11, D12, D13, D14, and D15.

### Assessment of vulvar swelling

To evaluate vulvar swelling, measurements of vulvar length and width were obtained on each observation day using a sterile, calibrated caliper. Before measurement, visual inspection was conducted for signs of estrus such as swelling and reddening of the vulva. Measurements commenced once the animal was properly restrained and calm, with the caliper care-fully placed at the outermost region of the vulva and adjusted until it made contact with the tissue.

### Cervical mucus collection and viscosity evaluation

Cervical mucus samples were collected in conjunction with vulvar assessments, following the onset of mucus discharge. The perineal region was cleaned with 70% alcohol and dried with sterile cotton. A sterile 20 mL pipette, affixed to a rubber hose, was inserted gently into the vaginal canal, and mucus was aspirated by palpation. Mucus viscosity was evaluated using the *spinnbarkeit* method as described by Tsiligianni *et al*. [16]. In this procedure, 2–3 drops of mucus were placed between two fat-free glass slides, which were then gradually separated. The stretch length of the mucus strand at the point of breakage was measured using a vernier caliper and recorded in millimeters.

### Measurement of VER

VER, expressed in Ohms (Ω), was measured using the Ovate instrument (Heritage Genetics, LLC, USA), consisting of a stainless-steel detachable probe and a battery-powered digital unit. The probe was disinfected and calibrated daily. Before insertion, the vulvar area was cleaned with a paper towel. The probe was gently introduced into the vaginal canal and rotated 4–5 times with slight back-and-forth movement until a stable reading was achieved. After each measurement, the probe was cleaned using a scrub pad, washed thoroughly, and disinfected with undiluted chlorhexidine followed by immersion in 0.03% chlorhexidine solution. It was rinsed with water and dried before the next use.

### Vaginal cytology evaluation

Vaginal epithelial cells were collected using sterile cotton swabs gently rotated inside the vaginal canal. The collected material was smeared onto pre-treated glass slides fixed with methanol. Samples were stained using the Romanowsky technique with Giemsa stain for 10–15 min, rinsed, air-dried, and examined microscopically at 400× magnification. For each smear, 100 epithelial cells were classified into parabasal, intermediate, superficial, and keratinized (cornified) types. Percentages were calculated for each category to characterize the cytological profile across the estrus cycle.

### Reproductive hormone profiling

Blood samples (1.5 mL) were collected from the jugular vein into vacutainer tubes without antico-agulant. After clotting, samples were centrifuged at 3500 rpm for 10 min, and serum was stored at –20°C until analysis. Concentrations of follicle-stimulating hormone (FSH), progesterone, and estradiol were determined using commercial enzyme-linked immunosorbent assay kits (DRG Instruments, GmbH, Germany), following the manufacturer’s instructions.

### Statistical analysis

Data were analyzed using GraphPad Prism version 10 (GraphPad Software, La Jolla, CA, USA). Results are presented as mean ± standard deviation. To evaluate differences across observation days for each parameter, one-way analysis of variance was conducted, followed by Tukey’s *post hoc* test. Statistical significance was set at p < 0.05.

## RESULTS

### Vulvar morphological changes

The external appearance of the vulva was used as an indicator of estrus, with vulvar swelling assessed by measuring the length and width of the vulvar fissure. The findings revealed a statistically significant increase in vulvar length on D14 following the initial PGF2α injection (p < 0.05), while vulvar width did not differ significantly across the days of observation (p > 0.05) (Table 1).

**Table 1:** Vulva size in Pasundan heifers during estrus synchronization.

Day	Mean ± SD (cm)	

	Length	Width
D0	6.13 ± 1.36^a^	4.16 ± 0.57
D4	6.67 ± 1.05^ab^	4.51 ± 0.65
D5	6.35 ± 1.05^ab^	4.35 ± 0.67
D11	6.08 ± 0.95^a^	4.15 ± 0.58
D12	6.79 ± 1.13^ab^	4.35 ± 0.64
D13	6.86 ± 1.31^ab^	4.39 ± 0.56
D14	7.27 ± 1.15^b^	4.56 ± 0.67
D15	6.62 ± 0.89^ab^	4.22 ± 0.65

Different superscripts in the same column indicate a statistically significant difference (p < 0.05). SD=Standard deviation

### Cervical mucus viscosity

One of the most prominent indicators of estrus was the alteration in the viscosity of vaginal mucus, observed during the synchronization protocol involving double injections of PGF2α.

**Figure 1:**
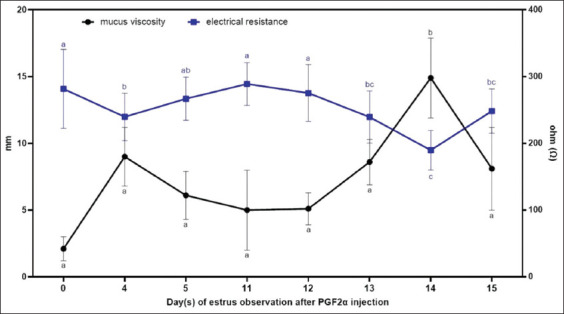
Vaginal mucus viscosity (black line) and vaginal electrical resistance (blue line) in Pasundan heifers during estrus synchronization. Values with different superscripts in the same line color indicate a statistical difference (p < 0.05).

Vaginal mucus exhibited the lowest viscosity on D0 and D11, corresponding to the administration of the first and second PGF2α injections, respectively (Figure 1, black line). Viscosity values increased from 2.10 ± 0.90 mm at D0 to 9.00 ± 2.20 mm at D4, followed by a gradual decline to 5.00 ± 3.00 mm, 5.10 ± 1.20 mm, and 8.60 ± 1.70 mm on D11, D12, and D13, respe-ctively. These changes were not statistically significant (p > 0.05). However, a marked increase in viscosity was noted following the second PGF2α dose, with the highest value recorded on D14 (14.9 ± 3.00 mm; p < 0.05). On D15, the viscosity sharply declined to 8.1 ± 3.10 mm.

### VER

Figure 1 (blue line) illustrates changes in VER during estrus synchronization. The mean VER values were signif-icantly different between D4 (239.67 ± 35.59 Ω) and D14 (198.67 ± 29.61 Ω), as compared to D0, D11, and D12, which exhibited higher readings of 281.67 ± 59.09 Ω, 289.00 ± 31.99 Ω, and 275.44 ± 42.45 Ω, respectively (p < 0.05). After the initial PGF2α injection, VER dec-lined from 281.76 ± 59.09 Ω on D0 to 266.89 ± 2.35 Ω on D5 then rose slightly on D11. A sharp reduction was observed on D14 (198.67 ± 29.61 Ω; p < 0.05) after the second PGF2α injection, followed by an increase to 248.33 ± 33.21 Ω on D15. The lowest mean VER value was recorded on D14.

### Vaginal cytology dynamics

**Figure 2:**
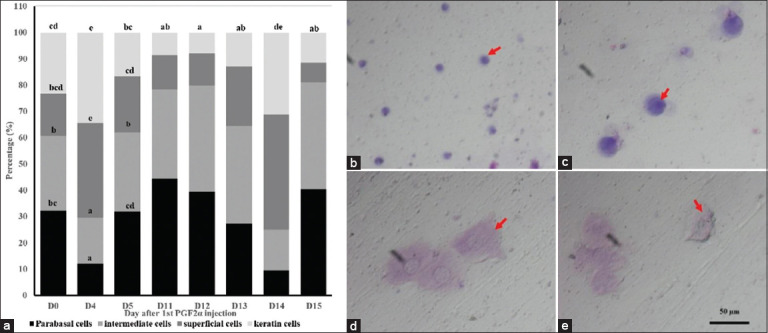
(a) Proportion of cytological cells in Pasundan heifers during the estrus period. (b) Epithelial cells at parabasal, round, small cells with a large nucleus. (c) Intermediate cells, large round cells with a large nucleus. (d) Superficial cells, polygonal cells with a large nucleus. (e) keratin cells, polygonal cells with a small or no nucleus. In the bar diagram, different superscripts in the same type of cells indicate a statistical difference (p < 0.05), red arrows represent vaginal mucosa epithelial cells.

The proportion of vaginal epithelial cell types during the estrus observation period is presented in Figure 2a. The cell types, such as parabasal, intermedi-ate, superficial, and keratinized are shown in Figures 2b–e. On D4, vaginal cytology was dominated by superficial and keratinized cells, accounting for 36.12% and 34.35% of the population, respectively. Both cell types declined sharply by D5 to 21.44% and 16.57%. After the second PGF2α injection on D11, parabasal (44.47%) and intermediate cells (33.84%) were predominant. Parabasal cells decreased progressively, reaching their lowest value on D14 (9.60%). Intermediate cells predominated on D12 (40.50%) and D13 (36.98%), while superficial cells increased to 12.28% and 22.48% on those respective days. These superficial cells continued to rise, peaking on D14, when the epithelium was again dominated by superficial and keratinized cells. On D15, both superficial and keratinized cells declined sharply, replaced by increasing proportions of parabasal and intermediate cells (Figure 2a).

### Reproductive hormone profiles

**Figure 3:**
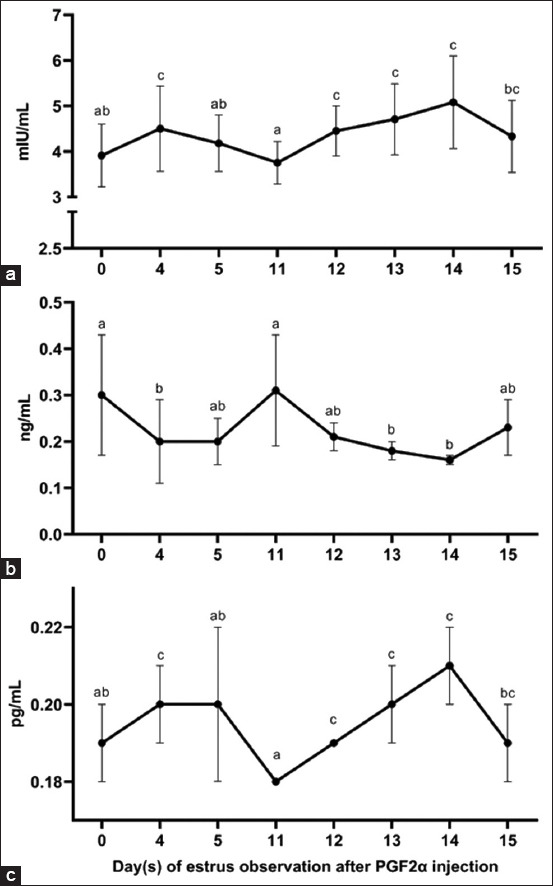
Hormone levels of (a) follicle-stimulating hormone, (b) progesterone, (c) and estradiol during estrus synchronization in Pasundan heifers. The different superscripts indicate a statistical difference (p < 0.05).

The hormonal profiles of FSH, progesterone, and estradiol across the observation period are shown in Figure 3. FSH levels (Figure 3a) were significantly elevated on D4, D5, D12, D13, and D14 (4.50, 4.18, 4.45, 4.71, and 5.08 mIU/mL, respectively) compared to D0 and D11 (3.91 and 3.75 mIU/mL; p < 0.05). FSH increased steadily from D11, peaked at D14, and declined to 4.33 mIU/mL on D15.

Progesterone levels (Figure 3b) showed a progressive decline from 0.314 ng/mL on D11 to 0.214 ng/mL on D12, 0.183 ng/mL on D13, and a nadir of 0.162 ng/mL on D14. This decline followed the second PGF2α injection. On D15, progesterone levels rose again to 0.227 ng/mL.

Estradiol levels (Figure 3c) were signif-icantly elevated on D4 (0.200 pg/mL) and D14 (0.214 pg/mL). From 0.183 pg/mL on D11, estradiol increased to 0.190 pg/mL on D12 and 0.198 pg/mL on D13, peaking at 0.214 pg/mL on D14 before decreasing to 0.189 pg/mL on D15. The D14 peak was statistically significant (p < 0.05) compared with all other time points.

### Estrus expression rate

Table 2 presents the number and percentage of Pasundan heifers displaying estrus signs during the synchronization period. On the day of the initial PGF2α injection (D0), a small number of heifers (16.67%) exhibited estrus. This proportion increased to appro-ximately 44.44% on D4. No estrus was observed on D11 following the second PGF2α injection. Estrus signs reappeared on D13 and peaked dramatically on D14 when 88.89% of the heifers showed observable estrus. No heifers were in estrus on D15.

**Table 2:** Number and proportion of Pasundan heifers in estrus during synchronization program.

Day	Estrus (n)	Percentage
D0	3	16.67
D4	8	44.44
D5	4	22.22
D11	0	0.00
D12	0	0.00
D13	2	11.11
D14	14	88.89
D15	0	0.00

## DISCUSSION

### Effectiveness of estrus synchronization in Pasundan heifers

Estrus synchronization is widely recognized for its ability to enhance the consistency of breeding and calving schedules, particularly benefiting beef cow-calf production systems. Cattle exhibit discernible behavioral cues during estrus [33], alongside changes in physiological parameters such as body temperature, vaginal cyto-logy [17], and reproductive hormone levels [18]. In this study, Pasundan heifers demonstrated sign-ificant changes in physical, cytological, and hormonal parameters on days 4 and 5 following the initial PGF2α injection.

### Early estrus indicators following first injection

On D4, vulvar hypertrophy was observed, accom-panied by increased vaginal mucus viscosity. This finding aligns with the previous study by Huang *et al*. [19] indicating that elevated estradiol concentrations during the follicular phase are associated with vulvar swelling and enhanced vaginal secretions. These outcomes correspond with our observations, particularly the elevated estradiol levels on D14 (Figure 3c), and are consistent with vulvar swelling (Table 1) and increased mucus viscosity (Figure 1). Estradiol stimulates the thickening and vascularization of the vaginal epithelium, producing swollen, reddish genitalia and abundant mucus secretion, which facilitates sperm transport and directly influences sperm function in the female reproductive tract [20, 21].

Concurrently, a decrease in VER was recorded on D4. This reduction is indicative of the presence of a mature follicle and correlates with the lutei-nizing hormone surge, which is a hallmark of estrus onset [22, 23]. VER reduction is likely due to increased hydration and vascular congestion in the vaginal mucosa, closely associated with estrus behavior and regulated by increasing estradiol and declining progesterone concentrations [24]. As supported by our findings, the lowest VER value appears to be a reliable indicator of estrus peak.

### Cytological shifts and hormonal dynamics

Estrus-related cytological changes were evident, mirroring those observed in vulvar size, mucus visc-osity, and VER. On D0, parabasal and intermediate cells were predominant, whereas on D4, a shift toward superficial and keratinized cells was noted. This cytological profile is consistent with the estrus phase and is associated with elevated estrogen levels that increase uterine activity, epithelial keratinization, and mitotic proliferation [25]. Keratinization further serves as a protective adaptation against microbial invasion [26]. Simultaneously, FSH and estradiol levels rose significantly on D4, while progesterone declined (Figure 3), indicating a transition into the follicular phase.

### Estrus onset variation post-first injection

The inconsistent estrus signs on D4 and D5 can be attributed to the heifers being at different stages of their ovarian cycles at the time of the first PGF2α injection. Heifers in the luteal phase responded to PGF2α by undergoing corpus luteum regression and hormone release, leading to estrus onset. In contrast, those in the follicular phase did not exhibit an immediate estrus response. The second PGF2α injection, administered on D11, was expected to synchronize all heifers into the luteal phase, promoting uniform estrus onset. PGF2α acts as a potent luteolytic agent, inducing vasocon-striction and subsequent regression of the corpus luteum [27].

### Peak estrus synchronization and hormone fluctuation post-second injection

Following the second injection, increases in vulvar size (Table 1) and mucus viscosity (Figure 1) were observed, culminating in D14, with a sharp decline in D15. This trend corresponded with the lowest VER recorded on D14 and subsequent cytological changes characterized by a dominance of superficial and keratinized epithelial cells (Figure 2a). By D15, these cell types diminished, with a resurgence of parabasal and intermediate cells, reflecting a return to a non-estrus state.

The observed vulvar swelling and reddening are attributable to increased vascular perfusion driven by hormonal oscillations during estrus [20]. Elevated FSH concentrations, indicative of follicular development, were evident after the second PGF2α injection, peaking on D14 (Figure 3a). This finding is supported by prior studies showing that FSH levels progressively increase from D11 to D14, corresponding to follicular maturation [28]. The dominant follicle on D14 likely secretes estradiol, which plays a key role in folliculogenesis and granulosa cell activity during the follicular phase [29]. High estradiol levels may also drive a pre-ovulatory FSH surge to support final follicle maturation [30]. Estradiol peaks just before ovulation, prompting estrus behavior and priming the reproductive system for potential conc-eption [31]. Following ovulation, the ruptured fol-licle differentiates into the corpus luteum, initiating progesterone secretion. Progesterone subsequently suppresses FSH through negative feedback, sustaining the early luteal phase and preparing the uterus for pregnancy [32].

## CONCLUSION

This study demonstrated that estrus synchronization using a double-injection protocol of PGF2α effectively induced uniform and physiologically consistent estrus responses in Pasundan heifers. Peak estrus occurred on day 14 post-initial injection, as evidenced by maximal vulvar swelling, highest cervical mucus viscosity (14.9 ± 3.00 mm), lowest VER (198.67 ± 29.61 Ω), and cytological dominance of superficial and keratinized cells. Concurrently, serum levels of estradiol and FSH peaked, while progesterone reached its nadir, supporting the reliability of these biomarkers for determining optimal insemination timing.

From a practical perspective, these findings provide a validated protocol for FTAI in Pasundan cattle, with day 14 identified as the optimal insemination window. This has substantial implications for improving reproductive efficiency and supporting conservation strategies for this genetically valuable but declining indigenous breed.

The study’s strength lies in its integrative assess-ment of estrus indicators – including morphological, cytol-ogical, and endocrine markers – across multiple synchronized timepoints, which enhances the precision of estrus detection and synchronization efficiency. Furthermore, the study was conducted under field-rele-vant conditions, increasing the applicability of results to practical herd management settings.

However, the study is limited by its relatively small sample size (n = 18) and the absence of fertility outcome data, such as conception or pregnancy rates following FTAI. In addition, variability in individual ovarian cycle status at the time of first PGF2α injection may have influenced the uniformity of initial estrus responses.

Future studies should aim to validate these findings on larger populations and assess pregnancy outcomes following FTAI. Comparative evaluations of alternative synchronization protocols, including GnRH-based regimens, could further refine reproductive management strategies. Longitudinal monitoring of reproductive performance and offspring quality will also be critical for optimizing herd-level genetic improv-ement programs.

## AUTHORS’ CONTRIBUTIONS

RW, NH, DR, RH, IH, and ARD: The study was developed, the experiment was carried out, and the data were curated. KRGA and MSB: Assessed the vaginal mucus cytology. SS: The hormonal assessment was performed. RS, SP, and VW: Data interpretation, statistical analysis, and graph presentation were performed. RW and SP: The original manuscript and revision were written. All authors have read and approved the final manuscript.
